# Integration of Metabolomics and Transcriptomics to Reveal the Antitumor Mechanism of *Dendrobium officinale* Polysaccharide-Based Nanocarriers in Enhancing Photodynamic Immunotherapy in Colorectal Cancer

**DOI:** 10.3390/pharmaceutics17010097

**Published:** 2025-01-13

**Authors:** Shengchang Tao, Huan Wang, Qiufeng Ji, Yushan Yang, Gang Wei, Ruiming Li, Benjie Zhou

**Affiliations:** 1 Department of Pharmacy, The Seventh Affiliated Hospital, Sun Yat-sen University, Shenzhen 518107, China; taoshch@mail.sysu.edu.cn (S.T.); jiqiufeng@sysush.com (Q.J.); yangyushan@sysush.com (Y.Y.); 2 Shenzhen Key Laboratory of Chinese Medicine Active Substance Screening and Translational Research, The Seventh Affiliated Hospital, Sun Yat-sen University, Shenzhen 518107, China; 3 School of Pharmaceutical Sciences, Guangzhou University of Chinese Medicine, Guangzhou 510006, China; wanghuan77777@outlook.com (H.W.); weigang021@outlook.com (G.W.)

**Keywords:** *Dendrobium officinale* polysaccharide, photodynamic therapy, metabolomics, transcriptomics, antitumor mechanism

## Abstract

**Background**: The mechanism of *Dendrobium officinale* polysaccharide-based nanocarriers in enhancing photodynamic immunotherapy in colorectal cancer (CRC) remains poorly understood. **Methods**: The effects of TPA-3BCP-loaded cholesteryl hemisuccinate–*Dendrobium officinale* polysaccharide nanoparticles (DOP@3BCP NPs) and their potential molecular mechanism of action in a tumor-bearing mouse model of CRC were investigated using non-targeted metabolomics and transcriptomics. Meanwhile, a histopathological analysis (H&E staining, Ki67 staining, and TUNEL assay) and a qRT-PCR analysis revealed the antitumor effects of DOP@3BCP NPs with and without light activation. **Results**: Through metabolomics and transcriptomics analysis, we found an alteration in the metabolome and functional pathways in the examined tumor tissues. The metabolic analysis showed 69 and 60 differentially expressed metabolites (DEMs) in positive- and negative-ion modes, respectively, in the treated samples compared to the Control samples. The transcriptomics analysis showed that 1352 genes were differentially expressed among the three groups. The differentially regulated functional pathways were primally related to the antitumor immune response. The results of the pathological histology assay and qRT-PCR analysis verified the findings of the integrated metabolomics and transcriptomics analysis. **Conclusions**: Overall, our findings elucidate the potential antitumor mechanisms of the *D. officinale* polysaccharide-based nanocarrier in enhancing photodynamic immunotherapy in CRC.

## 1. Introduction

Colorectal cancer (CRC) is the second-leading cause of mortality and has the third highest incidence rate of all cancers worldwide. With the utilization of advanced profiling technologies, we can further understand and treat this pernicious disease [1,2]. Surgery, chemotherapy, targeted therapy, and immunotherapy are the common treatment methods [3]. What cannot be ignored are the side effects of these methods, for example, organ toxicity, painfulness, and worsened prognosis [3,4]. Photodynamic therapy (PDT) is a promising potential treatment for CRC, with the advantage of being a non-invasive approach allowing for a precise spatiotemporal control, with adjustable photoactivity and minimal side effects [5,6,7,8].

We previously successfully synthesized new polysaccharide-based nanoparticles (NPs) through self-assembling with a positively charged TPA-3BCP photosensitizer (PS), named TPA-3BCP-loaded cholesteryl hemisuccinate–*Dendrobium officinale* (*D. officinale*) polysaccharide nanoparticles (DOP@3BCP NPs) [9]. *D. officinale* polysaccharides (DOPs) were extracted from *D. officinale*, which is a rare Chinese traditional herb, and were shown to possess good immunomodulatory activity [10]. To the best of our knowledge, natural polysaccharides like chitosan and hyaluronic acid have been widely applied in the development of nanomedicine, due to their notable properties such as biocompatibility, low toxicity, broad source, low cost, and facile modification [11,12,13]. Data from our studies suggested that DOP@3BCP NP-mediated PDT can significantly suppress the growth of CRC tumors and enhance the photodynamic immunotherapy of tumors through the promotion of dendritic cell (DC) maturation and antigen presentation [9]. In addition, we noticed that DOP@3BCP NPs exhibited good biocompatibility and caused almost no severe damage to the function of the liver and kidney. DOP@3BCP NPs, as a promising CRC therapeutic agent, have been reported to play an important role in enhancing tumor immunity [9], but the related downstream signaling mechanism remains unclear.

Multi-omics approaches combining massive datasets have recently been increasingly used to reveal the complex and convoluted molecular mechanisms underlying various diseases [14,15,16,17]. Currently, metabolomics can be applied to analyze various biological samples, such as plasma, serum, urine, tissue, saliva, and stool samples, which can reflect metabolic characteristics at the systemic circulation level [18]. Transcriptomics reflects the expression and regulation of genes in cells or tissues at the overall level, allowing for the elucidation of the molecular mechanisms of complex diseases [19,20]. The integration of metabolomics and transcriptomics has been applied to identify and reveal the changes in both metabolites and genes in various biological samples, demonstrating their efficacy as tools to enable a better understanding of the regulating mechanisms of diseases [21,22].

Thus, the main objective of this study was to explore the antitumor mechanisms of DOP@3BCP NPs after illumination through metabolomic and transcriptomic approaches. In addition, a qRT-PCR analysis further verified the expression of related cytokines. The results elucidated the potential key chemokines and pathways that may play a decisive role in the treatment of CRC by PDT.

## 2. Materials and Methods

### 2.1. Materials and Reagents

Cholesteryl hemisuccinate (CHS) was purchased from Shanghai Yuanye Bio-Technology Co., Ltd. (Shanghai, China). 1-(3-dimethyl aminopropyl)-3-ethylcarbodiimide hydrochloride (EDC⋅HCl) and 4-bromobenzene acetonitrile were purchased from Macklin Biochemical Co., Ltd. (Shanghai, China). Dimethyl aminopyridine (DMAP) was purchased from Bide Pharmatech Ltd. (Shanghai, China). Pyridine 4-boronic acid, dimethyl sulfoxide (DMSO), and 4,4′,4″-nitro trityl carboxaldehyde were purchased from Sigma Aldrich Co. (St. Louis, MO, USA). Potassium carbonate (K_2_CO_3_), bromoethane (C_2_H_5_Br), N,N-dimethylformamide (DMF), and piperidine were purchased from Aladdin Biotechnology (Shanghai, China). Tetrakis (triphenylphosphine) palladium was purchased from Innochem Ltd. (Beijing, China). Tetrahydrofuran (THF), methanol, ethanol, dichloromethane (CH_2_Cl_2_), petroleum ether, and ethyl acetate were purchased from Guangzhou Chemical Reagent Factory (Guangzhou, China).

Phosphate-buffered saline (PBS) was purchased from Biosharp Biotechnology (Hefei, Anhui, China). Fetal bovine serum (FBS) was purchased from Priscilla Biotechnology Co., Ltd. (Wuhan, Hubei, China). Dulbecco’s modified Eagle’s medium (DMEM), penicillin, streptomycin, and 0.25% EDTA/trypsin were purchased from Gibco Invitrogen Co. (Grand Island, NY, USA). DNase/RNase-free water was purchased from Solarbio (Beijing, China). The PDT experiments used a GR-60WL cold white light source (400–680 nm, Minebea Co., Ltd., Tokyo, Japan) as in a previous study [9].

### 2.2. Preparation of DOP@3BCP NPs

We synthesized the TPA-3BCP-loaded cholesteryl hemisuccinate–*D. officinale* polysaccharide nanoparticles (DOP@3BCP NPs) according to our previous study [9], the details of which are shown in Supplementary Information.

### 2.3. Cells and Animals

CT-26 murine colon cancer cells were acquired from the Cell Line Bank of the Chinese Academy of Sciences (Shanghai, China). The cells were cultured in complete DMEM containing 10% FBS, supplemented with 1% penicillin/streptomycin, and incubated at 37 °C in a 5% CO_2_ atmosphere. The cells were passaged every 2–3 days according to cell density. BALB/c mice (female, 4–6 weeks old) were purchased from Guangdong Zhiyuan Biomedical Technology Co., Ltd. (Guangzhou, China) and fed in a specific-pathogen-free animal facility. All the animal experimental procedures were conducted in strict accordance with the guidelines of the Animal Ethics Committee of Guangzhou University of Chinese Medicine.

The CT-26 subcutaneous tumor model was prepared by injecting 5 × 10^6^ CT-26 cells into the right hind legs of the BALB/c mice. When the tumor volume reached 60–80 mm^3^, the mice were randomly divided into three groups of five mice each and maintained on a 12 h light/dark cycle. The three groups were named Control (PBS), DOP (DOP@3BCP NPs), and DOPL (DOP@3BCP NPs + L; the letter L indicates the use of light activation). During the treatment, the mice in the DOP and DOPL groups were administered DOP@3BCP NPs (10 mg/kg per mouse of DOP@3BCP NPs), and those in the Control group were administered a corresponding volume of PBS via direct injection into the tumor on days 0 and 3. In addition, after the administration of treatment, the DOPL group received extra irradiation with white light (150 mW/cm^2^) for 10 min (using a 1 min interval every 2 min to prevent a heating effect). After treatment, the mice were euthanized via cervical spine dislocation, and all tumor samples were collected and kept frozen at −80 °C or fixed with 4% polyoxymethylene for further analysis.

### 2.4. Non-Targeted Metabolomic Analysis Based on UPLC-ESI-MS/MS

Metabolite profiling was performed on 100 mg of tumor tissue from 15 samples using a UPLC system (Shim-pack UFLC SHIMADZU CBM30A, Shimadzu, Kyoto, Japan) coupled with an ESI–MS/MS system (Applied Biosystems 4500 Q TRAP, AB SCIEX, Foster City, CA, USA) with an orthogonal electrospray ionization (ESI) source under positive- and negative-ionization modes. The chromatographic separation was performed using a Waters ACQUITY UPLC HSS T3 C18 column (2.1 × 100 mm, 1.8 µm particle diameter, Waters, Milford, MA, USA) at 40 °C. The mobile phases were composed of H_2_O with 0.1% formic acid (A) and acetonitrile with 0.1% formic acid (B), and the elution gradient program was as follows: 5–90% B for 0–11 min, 90% B for 11–12 min, 90–5% B for 12–12.1 min, and 5% B for 12.1–14 min. The flow rate was 0.40 mL/min, and the sample injection volume was 2 µL. The mass spectrometer used the following parameters: source temperature and sheath temperature, 325 °C; capillary voltage, 2500 V (ESI+) and 1500 V (ESI−); and curtain gas and ion source gas I and II set at 25, 55, and 60 psi, respectively. The collision gas was set to high.

Supervised partial least-squares discriminant analysis, principal component analysis (PCA), hierarchical cluster analysis (HCA), and orthogonal partial least-squares discriminant analysis (OPLS-DA) were conducted to discriminate the Control and the drug-treated groups using R software (version 3.5.1, 1.2.1, 2.71.1009, and 1.0.1). Significant differences in metabolite expression among the different groups were determined based on the screening criteria of variable importance in the projection (VIP) > 1 and *p*-value < 0.05, to finally identify the differentially expressed metabolites (DEMs). Meanwhile, the identified metabolites were annotated using the Kyoto Encyclopedia of Genes and Genomes (KEGG) Compound database (http://www.kegg.jp/kegg/compound/, accessed on 24 August 2024), and the metabolic pathways were enriched by mapping to the KEGG Pathway database (http://www.kegg.jp/kegg/pathway.html, accessed on 24 August 2024). All analysis was performed using the Metware Cloud platform (https://cloud.metware.cn).

### 2.5. Transcriptome Sequencing

Following the manufacturer’s protocol, total RNA was extracted using the MJZol total RNA extraction kit (Shanghai, China) from tumor tissue of mice in the Control, DOP, and DOPL groups (*n* = 3, randomly). Then, the samples were sent to the Shanghai Majorbio Bio-pharm Biotechnology Co., Ltd. (Shanghai, China), where RNA sequencing was performed on an Illumina NovaSeq Xplus platform (San Diego, CA, USA) with 2 × 150 bp read lengths. The identification, enrichment, and cluster analysis of the differentially expressed genes (DEGs) were further performed using the Majorbio cloud platform (https://www.majorbio.com/). The DEGs were identified by DESeq2 using |log2FoldChange| ≥ 1 and *p*-value < 0.05 as the threshold. Gene Ontology (GO, http://geneontology.org/), KEGG (https://www.genome.jp/kegg/, accessed on 2 August 2024), and Reactome (https://reactome.org/) functional and enrichment analyses for DEGs were performed using GOATOOLS (https://github.com/tanghaibao/GOatools, accessed on 2 August 2024) and SciPy (https://scipy.org/install/, accessed on 2 August 2024) [23,24].

### 2.6. H&E, Ki67, and TUNEL Staining

All tumors from the three groups were fixed with 4% polyoxymethylene for hematoxylin and eosin (H&E) staining, terminal deoxynucleotidyl transferase-mediated dUTP-biotin nick-end labeling (TUNEL) assay, and antigen Ki67 staining. H&E dye solution and a TUNEL assay kit were purchased from Servicebio Technology Co. (Wuhan, Hubei, China). Immunohistochemistry (IHC) for the Ki67 and TUNEL assays was performed according to the manufacturer’s protocols to investigate proliferation and apoptosis in the tumor tissues. The H&E-stained and IHC-stained tissue sections were examined using an optical microscope (Olympus BX53, Tokyo, Japan), and TUNEL staining was observed using a confocal laser scanning microscope (CLSM, Nikon A1, Nikon Corporation, Tokyo, Japan).

### 2.7. Quantitative Real-Time PCR Analysis

Total RNA was extracted from the tumor tissue using the FastPure Cell/Tissue Total RNA Isolation Kit (Vazyme Biotech, Nanjing, China), according to the manufacturer’s instructions. Then, RNA was reverse-transcribed into cDNA using specific primers selected on the basis of the transcriptome analysis, following the instructions provided with the All-in-One First-Strand Synthesis MasterMix (with dsDNA) Reagent Kit (Guangzhou Xinkailai Biotechnology Co., Ltd., Guangzhou, China) and then stored at −80 °C for future use. GAPDH was used as an internal reference. The primers were synthesized by Sangon Biotech Co., Ltd. (Shanghai, China). Quantitative real-time PCR (qRT-PCR) was carried out using the SYBR Green Premix (Universal) (Guangzhou Xinkailai Biotechnology Co., Ltd., Guangzhou, China) and a qTOWER^3^ G Real-Time PCR thermal cycler (Analytik Jena, Jena, Germany). The 2^−ΔΔCt^ method was used to determine the relative gene expression levels. The primer sequences used are shown in Table 1.

### 2.8. Statistical Analysis

All the results are expressed as the means ± standard deviations (SDs) of three separate experiments. GraphPad Prism 10.1.2 (GraphPad Software, La Jolla, CA, USA) was used to analyze and visualize the parameters. The data were analyzed using one-way ANOVA, followed by Dunnett’s post hoc test using the SPSS version 26.0 program (IBM SPSS Inc., New York, NY, USA); *p* < 0.05 was considered to indicate statistical significance.

## 3. Results

### 3.1. Synthesis and Characterization of DOP@3BCP NPs

DOP@3BCP NPs are composed of two parts, amphiphilic CHS-DOP and TPA-3BCP. Their characterization was published in a previous report [9] and is described in the Supplementary Information S1 (Figure S1).

### 3.2. Effects of DOP@3BCP NPs on the Metabolome of the Tumors

**Figure 1:**
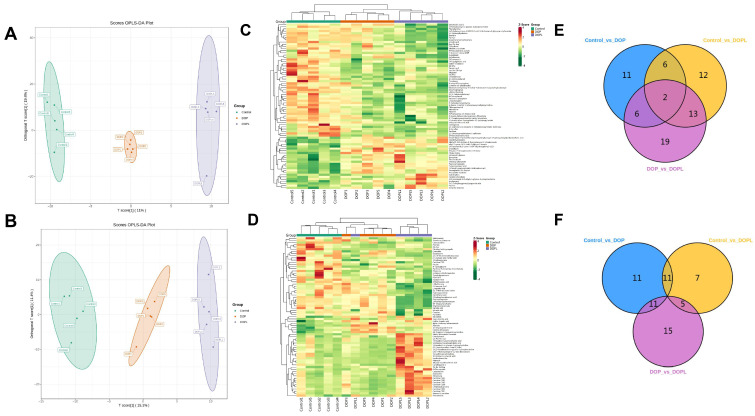
Effects of DOP@3BCP NP-mediated PDT on metabolite profiling of tumor among three groups. Score plot of OPLS-DA in positive ion mode (**A**) and in negative ion mode (**B**) from tumor tissue metabolomics data; Heatmap of DEMs in positive- (**C**) and negative-ion mode (**D**) from tumor tissue metabolomics data; Venn diagram of in positive- (**E**) and negative-ion mode (**F**) from tumor tissue metabolomics data.

To compare the metabolite composition of the three groups (Control, DOP, and DOPL), the datasets obtained from UPLC-ESI-MS/MS were subjected to OPLS-DA. The score plot results showed that the three groups were separated from each other (Figure 1A,B) based on the screening criteria of VIP > 1 and *p*-value < 0.05 for significantly different metabolite expression. In total, 1900 cationic metabolites and 2795 anionic metabolites were identified. In positive- and negative-ion modes, 69 and 60 DEMs were identified in the three groups, respectively, suggesting that DOP@3BCP NP-mediated PDT induced significant metabolomic diversification in CRC (Tables S1 and S2 in Supplementary Information S2).

Variations in the number of DEMs among the Control, DOP, and DOPL groups are visualized in the heatmap in Figure 1C,D. In positive-ion mode, compared to the Control, 25 (9 up- and 16 downregulated) and 33 (15 up- and 18 downregulated) DEMs were identified in DOP and DOPL, respectively (Figure 1E). Compared with the Control group, 33 and 23 DEMs were identified in DOP and DOPL in negative-ion mode, respectively (Figure 1F). In total, 18 and 15 of the former were down- and upregulated, while 11 and 12 of the latter were down- and upregulated, respectively. We found 31 and 40 DEMs in DOP vs. DOPL in the positive- and negative-ion modes, respectively. These particular DEMs indicated that after illumination, under the influence of PDT, reactive oxygen species (ROS) might be produced and kill tumor cells. This may further induce changes in metabolite expression. The identified DEMs were mainly organic acids and their derivatives; hormones; hormone-related compounds; heterocyclic compounds; benzene and substituted derivatives; aldehydes; ketones; esters; nucleotides and their metabolites; carbohydrates and their metabolites; fatty acids (FAs); glycerides (GLs); and glycerophospholipids (GPs). Bar charts of the top 20 DEMs are shown in Figure S2 (Supplementary Information S1).

### 3.3. Metabolic Pathway Analysis of DEMs

**Figure 2:**
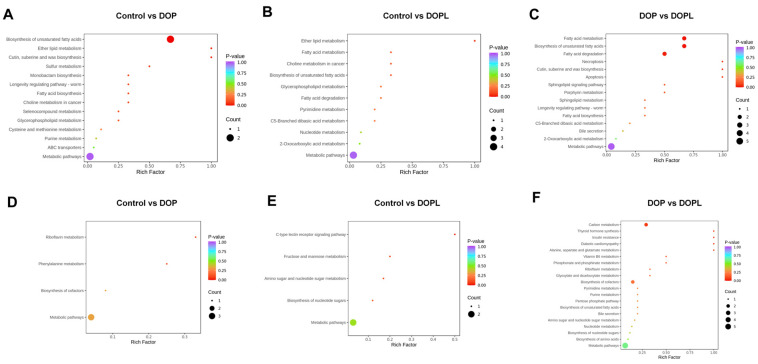
Bubble diagram of top 20 KEGG pathway enrichments in the positive- and negative-ion mode (*n* = 5): (**A**–**C**), in positive-ion mode; (**D**–**F**), in negative-ion mode.

To identify the biological pathways related to NP treatment with light exposure, the DEMs were mapped using the KEGG, and altogether, 40 and 29 metabolic pathways appeared to be affected in the positive- and negative-ion modes, respectively. The top 20 enriched pathways are displayed in Figure 2. Compared with the DOP group, the enriched pathways in the Control group mainly involved metabolism, including metabolic pathways, glycerophospholipid metabolism, ether lipid metabolism, choline metabolism in cancer, purine metabolism, cysteine and methionine metabolism, sulfur metabolism, riboflavin metabolism, and phenylalanine metabolism (Figure 2A,D). Similarly, the Control group, compared with the DOPL group, also showed some metabolism-related changes, but there were differences in the affected metabolic pathways, which included pyrimidine metabolism, nucleotide metabolism, C5-branched dibasic acid metabolism, glycerophospholipid metabolism, ether lipid metabolism, fatty acid metabolism, 2-oxocarboxylic acid metabolism, fructose and mannose metabolism, and amino sugar and nucleotide sugar metabolism. Notably, the results showed that more pathways affected in the DOP group were cancer-related pathways compared to the DOPL group, such as apoptosis and necroptosis, which might potentially mediate the effect of NPs in CRC.

### 3.4. Transcriptomic Analysis

**Figure 3:**
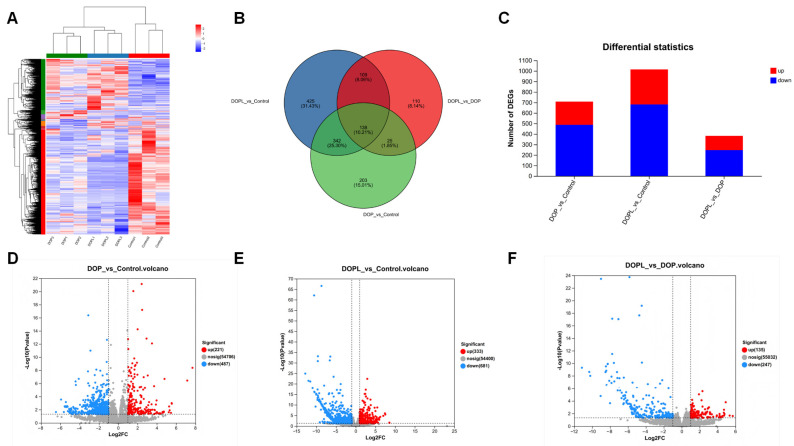
Profiling of DEGs in tumor tissues from different groups (*n* = 3). (**A**) Clustered heatmap for DEGs of different groups. (**B**) Venn diagram of DEGs from different groups. (**C**) Stacked bar plots for DEGs from different groups. (**D**–**F**) Volcano plot for DEGs from different groups.

To further determine the mechanism of action of DOP@3BCP NPs in CRC, we performed transcriptomic sequencing and analysis of DEGs in the tumor tissues after PDT. The screened DEGs are displayed in the heatmap, Venn diagram, stacked bar plots, and volcano plot in Figure 3, which shows that gene expression was significantly different in the three groups. A total of 1352 DEGs were identified across the three groups, and we found that some of them were closely related to antitumor immunity (Table S3 in Supplementary Information S2). Compared with the Control, DOP showed 221 and 487 up- and downregulated genes, respectively, and DOPL showed 333 and 681 up- and downregulated genes, respectively (Figure 3D,E). A total of 382 DEGs (135 up- and 247 downregulated) were identified when comparing DOPL and DOP (Figure 3F).

**Figure 4:**
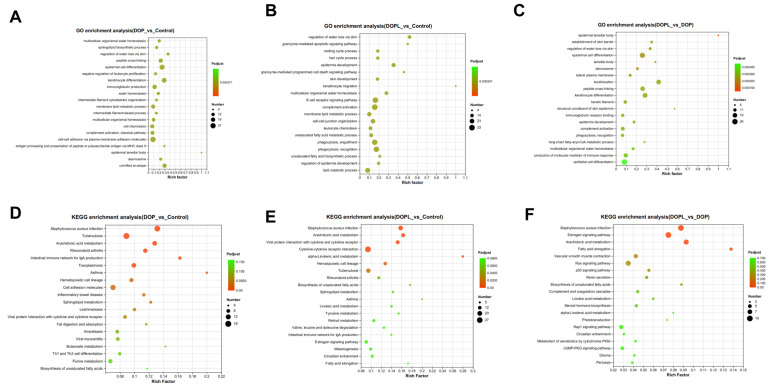
Functional enrichment of DEGs in tumor tissues from different groups (*n* = 3): (**A**–**C**)**,** GO functional enrichment analysis; (**D**–**F**)**,** KEGG functional enrichment analysis.

To further identify potential molecular functions affected by the DOP@3BCP NPs, the GO and KEGG databases were used to analyze the biological functions of the identified DEGs, and the 20 most significant signaling pathways were selected to draw a bubble chart (Figure 4). This chart indicates that the enriched molecular functions were mainly related to immunity and antitumor function in the DOP and DOPL groups compared to the Control group, suggesting that the antitumor mechanism of DOP@3BCP NP-mediated PDT may involve the immune response. Figure 4A,C show the top 20 GO terms enriched with the DEGs. We noticed that pathways like immunoglobulin production, antigen processing and presentation of peptide or polysaccharide antigens via MHC class II, the granzyme-mediated apoptotic signaling pathway, the granzyme-mediated programmed cell death signaling pathway, and the B cell receptor signaling pathway were enriched in the treated groups. In addition, after illumination (DOPL group), immunoglobulin receptor binding and the production of molecular mediators of the immune response were affected. The KEGG pathway enrichment analysis was consistent with the GO analysis (Figure 4D–F). The DEGs identified in the in the DOP and DOPL groups, compared to the Control, were involved in the intestinal immune network for IgA production, inflammatory bowel disease, and Th1 and Th2 cell differentiation. This suggests that polysaccharides may influence intestinal immunity. It is worth mentioning that the Ras and p53 signaling pathways, as far as we know, are closely related to apoptosis and were enriched in DOP vs. DOPL. The Reactome enrichment analysis is displayed in Figure S3 (Supplementary Information S1).

### 3.5. H&E, Ki67, and TUNEL Staining of Tumors

To gain deeper insights into the effects of the used nanoparticles (NPs) on cellular density, proliferation, and apoptosis within the tumors, H&E, Ki67, and TUNEL staining were performed on representative paraffin-embedded tumor sections.

**Figure 5:**
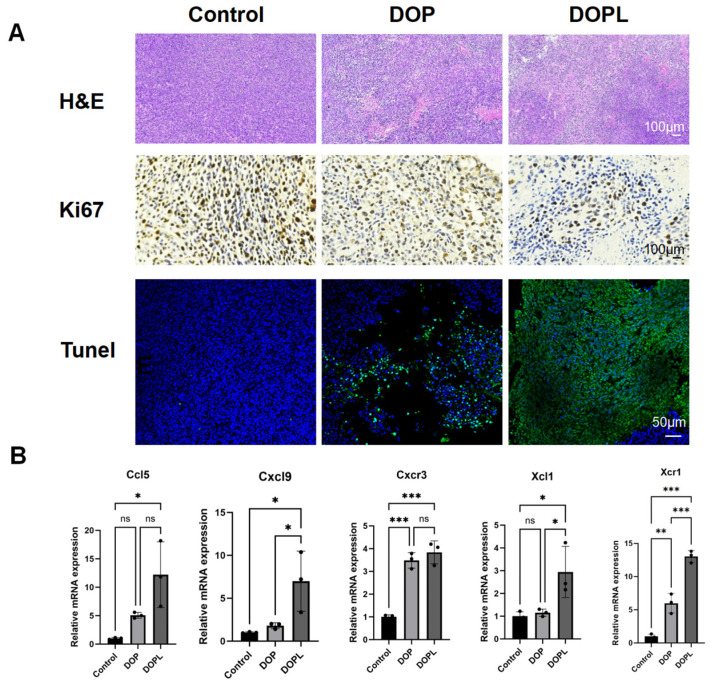
(**A**) Representative images of H&E, Ki67, and TUNEL staining of tumor slices in three groups. (**B**) Relative mRNA expression in the tumor was detected by real-time PCR, GAPDH served as a loading control. Data are expressed as means ± SD. * *p* < 0.05, ** *p* < 0.01, *** *p* < 0.001, and ns means no significance.

In H&E staining, hematoxylin stains the cell nuclei blue-purple, while eosin stains the cytoplasm and extracellular matrix pink. Damaged or apoptotic cells exhibit disrupted nuclei, which results in reduced blue–purple staining. As shown in Figure 5A, the DOP and DOPL groups exhibited markedly less blue–purple staining compared to the Control group, with the most significant reduction observed in the DOPL group, subjected to light exposure. This indicated substantial tumor cell damage, suggesting that the DOP@3BCP NPs, particularly in the presence of light, effectively enhanced tumor destruction. Ki67 immunohistochemical staining was used to assess cellular proliferation. Positive Ki67 signals appear as coarse brown–yellow granules localized in the cytoplasm and nuclei of proliferative cells, while negative cells exhibit blue-stained nuclei with no cytoplasmic or nuclear coloration. The DOP and DOPL groups showed reduced brown–yellow signals and increased blue staining compared to the Control group; this was particularly observed in the DOPL group. These findings indicate that the DOP@3BCP NPs significantly inhibited tumor cell proliferation, with the strongest effect observed under photodynamic activation. Moreover, to evaluate apoptosis, TUNEL staining was performed using DAPI (blue fluorescence) to label the nuclei and FITC (green fluorescence) to mark fragmented DNA, indicative of apoptosis. The co-localization of blue and green signals in the nuclei identifies apoptotic cells. The DOPL group exhibited the highest intensity of green fluorescence, corresponding to extensive DNA damage, thereby indicating enhanced tumor apoptosis following light activation.

Overall, these results collectively demonstrate that the DOP@3BCP NPs, particularly under light activation, significantly reduced tumor cell density, inhibited proliferation, and promoted apoptosis, which makes them a potent therapeutic candidate for photodynamic therapy.

### 3.6. Verification of the Gene Expression Levels

Based on the transcriptome analysis, five DEGs (Ccl5, Cxcl9, Cxcr3, Xcl1, and Xcr1) closely related to antitumor immunity and belonging to the chemokine factor family were selected to verify the reliability of the transcriptomic profiling data using qRT-PCR. Consistent with the transcriptomic results, the mRNA expression levels of the examined chemokines indicated that Ccl5, Cxcl9, Cxcr3, Xcl1, and Xcr1 expression increased significantly in the DOPL group compared with the Control group (Figure 5B). The DOPL group showed a clearer upregulated trend. These results further confirmed that the polysaccharide-based nanoparticles produced a corresponding antitumor response under light.

## 4. Discussion

CRC remains one of the leading causes of cancer-related deaths worldwide, and despite advances in its treatment, effective therapies are still urgently needed. PDT has shown promise in triggering immune responses that aid in the elimination of tumors, though the precise mechanisms underlying its anti-cancer effects remain poorly understood [5]. In our previous work, we demonstrated that DOP, a biocompatible carrier, enhances DC maturation, thereby improving the efficacy of photodynamic immunotherapy [9]. The goal of this study was to integrate metabolomic and transcriptomic data to explore the underlying molecular mechanisms of DOP@3BCP NP-mediated PDT in treating CRC. Our findings suggest that DOP@3BCP NP-mediated PDT altered the tumor metabolome and functional pathways associated with immune responses in a subcutaneous CRC tumor mouse model, exerting anti-cancer activity.

First, through metabolomic analysis, we identified 69 and 60 DEMs in the DOP and DOPL groups, respectively, compared to the Control group, with significant changes in metabolic pathways related to amino acid metabolism and unsaturated fatty acid biosynthesis. These metabolic shifts indicated that the DOP@3BCP NPs affected both the tumor microenvironment and immune cell metabolism. We observed that immune-related metabolic pathways, such as phenylalanine metabolism, the biosynthesis of unsaturated fatty acids, and the pentose phosphate pathway, were significantly altered in the treated groups. The involvement of these pathways in immune cell function and proliferation suggests that DOP@3BCP NP-mediated PDT may enhance immune responses through the modulation of metabolic pathways crucial for T cell activation and immune effector functions [25].

In terms of immunity, specific changes in metabolic pathways appear to directly impact the production of cytokines and the activation of immune cells. Our results are consistent with the growing body of literature indicating that immune cells rely on amino acid metabolism for energy and biosynthetic intermediates necessary for proliferation and activation [25]. In our study, DOP@3BCP NP-mediated PDT appeared to activate immune cells and promote antitumor immunity, particularly through the alteration of metabolic pathways that support immune cell functions.

Next, we performed transcriptomic analysis to better understand the gene expression changes induced by DOP@3BCP NP-mediated PDT in CRC. A total of 1352 DEGs were identified across the three groups (Control, DOP, and DOPL), with notable shifts in the expression of genes involved in immune response, apoptosis, and tumor progression. Our GO and KEGG analyses further highlighted several pathways related to immune regulation and antitumor responses. Specifically, we identified enrichment in pathways related to immunoglobulin production, antigen processing and presentation, and B cell receptor signaling, all of which are essential for effective antitumor immunity. The differential expression of genes related to unsaturated fatty acid biosynthesis was particularly notable, as unsaturated fatty acids play a critical role in cancer progression and have been implicated in cancer prevention and treatment [26].

Among the altered pathways, we also observed a significant modulation of key immune-related pathways, such as the intestinal immune network for IgA production and pathways related to inflammatory bowel disease. These pathways suggest that the DOP@3BCP NPs may also influence intestinal immunity, a crucial component of the body’s overall immune response. Interestingly, the Ras and p53 signaling pathways, both of which are intimately involved in apoptosis and cell survival, were enriched in the DOP group vs. the DOPL group, indicating that the nanoparticles may exert their anti-cancer effects through both immune activation and direct induction of tumor cell death.

Furthermore, our study identified a striking modulation of chemokine levels following PDT. Chemokines play a pivotal role in recruiting immune cells, such as CD8+ T cells and NK cells, to the tumor microenvironment (TME), where they can exert antitumor effects [27,28,29,30]. Chemokine axes, such as the CXCR3-CXCL9 and XCR1-CCL5 pathways, have been shown to enhance immune cell recruitment and promote antitumor immunity [31,32,33,34,35,36,37]. In line with these findings, our previous studies demonstrated that DOP@3BCP NP-mediated PDT stimulated CD8+ T cells and NK cells, further supporting the hypothesis that chemokine activation plays a key role in the immune response triggered by PDT [9]. The upregulation of chemokines following PDT, confirmed by qRT-PCR, suggests that DOP@3BCP NP-mediated PDT may recruit and activate immune cells and inhibit tumor growth, thus providing a potential mechanism for its therapeutic effects.

**Figure 6:**
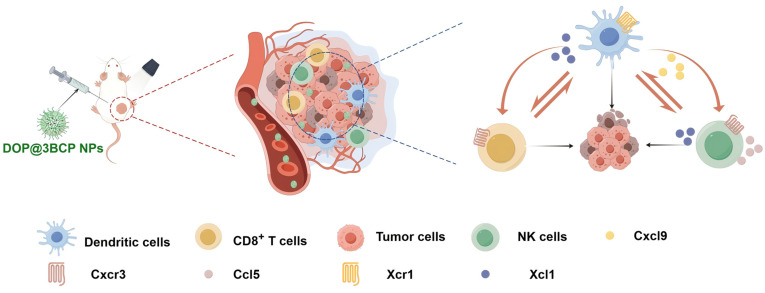
Schematic diagram illustrating the potential mechanism of DOP@3BCP NP-mediated PDT in CRC.

In summary, our integrated multi-omics analysis shed light on the complex molecular mechanisms underlying the anti-cancer effects of DOP@3BCP NP-mediated PDT. The findings indicate that in our model, the treatment modulated both metabolic and transcriptomic pathways, enhancing immune responses and inducing tumor cell apoptosis, offering valuable insights into the therapeutic potential of this strategy for CRC treatment (Figure 6).

## 5. Conclusions

In the current study, we integrated metabolomic and transcriptomic data to provide new insight into the molecular mechanism underlying the anti-CRC activity of DOP@3BCP NP-mediated PDT. We observed significant alterations in the metabolomic and functional pathways associated with their antitumor activity. We also verified the antitumor activity mechanisms via a pathological histology assay and qRT-PCR analysis. Overall, the results suggest that DOP@3BCP NP-mediated PDT can activate chemokines, thereby enhancing antitumor immunity. Altogether, this work provides new insights into the potential anti-CRC mechanisms of DOP@3BCP NP-mediated PDT, offering a promising paradigm in clinical cancer treatment.

## Figures and Tables

**Table 1:** The sequences of the primers used in qRT-PCR.

Gene Name	Forward (5′→3′)	Reverse (5′→3′)
GAPDH	GGCAAATTCAACGGCACAGT	AGATGGTGATGGGCTTCCC
Ccl5	GCATCCCTCACCGTCATCCTC	GCACTTGCTGCTGGTGTAAAA
Cxcl9	AATGCACGATGCTCCTGCA	AGGTCTTTGAGGGATTTGTAGTGG
Cxcr3	TACGATCAGCGCCTCAATGCCA	AGCAGGAAACCAGCCACTAGCT
Xcl1	CTTTCCTGGGAGTCTGCTGC	CAGCCGCTGGGTTTGTAAGT
Xcr1	AGAGACACCGAACAGTCAGGCT	TGTCCAGTTGCTGAAGGCTCTC

## Data Availability

All data generated or analyzed are included in this article. Transcriptomics data can be found in the National Centre for Biotechnology Information (NCBI) BioProject database under accession number PRJNA1190111.

## Supplementary Materials

The following supporting information can be downloaded at: https://www.mdpi.com/article/10.3390/pharmaceutics17010097/s1, Figure S1: Particle size distribution and morphology of DOP@3BCP NPs (scale bar = 100 nm); Figure S2: Bar chart of top 20 DEMs in the positive and negative ion mode (*n* = 5); Figure S3: Reactome functional enrichment of DEGs in tumor tissues from different groups (*n* = 3); Table S1: Significantly altered metabolites among three groups in positive ion mode; Table S2: Significantly altered metabolites among three groups in negative ion mode; Table S3: Significantly altered genes among three groups. References [9,10] are cited in the Supplementary Materials.
