# Supplementary material for: Integration of Metabolomics and Transcriptomics to Reveal the Antitumor Mechanism of Dendrobium officinale Polysaccharide-Based Nanocarriers in Enhancing Photodynamic Immunotherapy in Colorectal Cancer
Source: Pharmaceutics. 2025 Jan 13;17(1):97. doi: 10.3390/pharmaceutics17010097 (PMC11769008; doi:10.3390/pharmaceutics17010097)
Supplement: Supplementary file 1 [file pharmaceutics-17-00097-s001.zip › Supplementary Information S1.pdf]

Supplementary Information

Integration of Metabolomics and Transcriptomics to Reveal the Antitumor Mechanism of *Dendrobium officinale* Polysaccharide-Based Nanocarriers in Enhancing Photodynamic Immunotherapy in Colorectal Cancer

Shengchang Tao [1,2,†], Huan Wang [3,†], Qiufeng Ji [1,2], Yushan Yang [1,2], Gang Wei [3], Ruiming Li [1,2,*] and Benjie Zhou [1,2,*]

[1] Department of Pharmacy, The Seventh Affiliated Hospital, Sun Yat-sen University, Shenzhen 518107, China; taoshch@mail.sysu.edu.cn (S.T.); jiqiufeng@sysush.com (Q.J.); yangyushan@sysush.com (Y.Y.)
[2] Shenzhen Key Laboratory of Chinese Medicine Active Substance Screening and Translational Research, The Seventh Affiliated Hospital, Sun Yat-sen University, Shenzhen 518107, China
[3] School of Pharmaceutical Sciences, Guangzhou University of Chinese Medicine, Guangzhou 510006, China; wanghuan77777@outlook.com (H.W.); weigang021@outlook.com (G.W.)
* Correspondence: liruiming@sysush.com (R.L.); zhoubenjie@sysush.com (B.Z.)
† These authors contributed equally to this work.

Synthesis and characterization of DOP@3BCP NPs

Synthesis of DOP@3BCP NPs

In brief, we firstly extracted and oxidative-degrade, and purified *Dendrobium officinale* polysaccharide (DOP) follow the methods in accordance with a previous research, by which we obtained of DOP with average molecular weight 80.32 kDa [1]. DOP and cholesteryl hemisuccinate were used to synthesize amphiphilic cholesteryl hemisuccinate-*Dendrobium officinale* polysaccharide (CHS-DOP) at room temperature (R.T.) in a magnetic stirrer through the catalysis of EDC·HCl and DMAP. Next, we synthesized aggregation-induced emission (AIE) photosensitizers, named as TPA-3BCP, according to the previous method [2]. The conjunction of TPA-3BCP and CHS-DOP was made by slowly injecting TPA-3BCP/DMSO into CHS-DOP in water. After centrifuge, dialysis, and filtration, the DOP@3BCP NPs was finally assembled and synthesized.

Characterization of DOP@3BCP NPs

The particle size and Zeta potential of DOP@3BCP were measured by NanoBrook 90Plus Zeta (Brookhaven Instruments Corporation, New York, USA) and Nano-ZS90 ZetaSizer, respectively (Malvern Panalytical Ltd., UK). The morphology of DOP@3BCP was observed by a JEOL JEM-2100 transmission electron microscopy (TEM) (JEOL, Tokyo, Japan). Briefly speaking, the loading content and loading efficiency of TPA-3BCP in DOP@3BCP NPs were 14.19% and 85.18%, respectively. The results showed that DOP@3BCP NPs measured approximately 200 – 250 nm and +13.06 mV, respectively. The TEM caption illustrated the morphology of NPs was uniform and monodisperse (**Figure S1**).

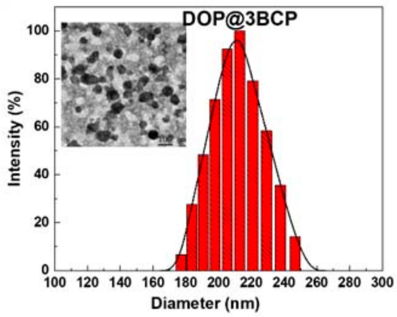

Figure S1. Particle size distribution and morphology of DOP@3BCP NPs (scale bar = 100 nm)

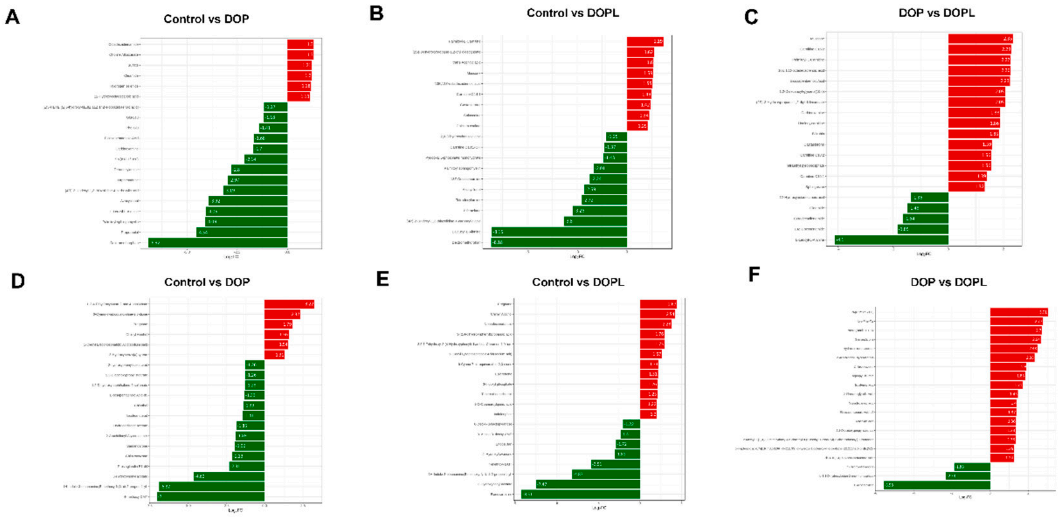

Figure S2. Bar chart of top 20 DEMs in the positive- and negative- ion mode (n =5). A, B, C, in positive-ion mode; D, E, F, in negative-ion mode.

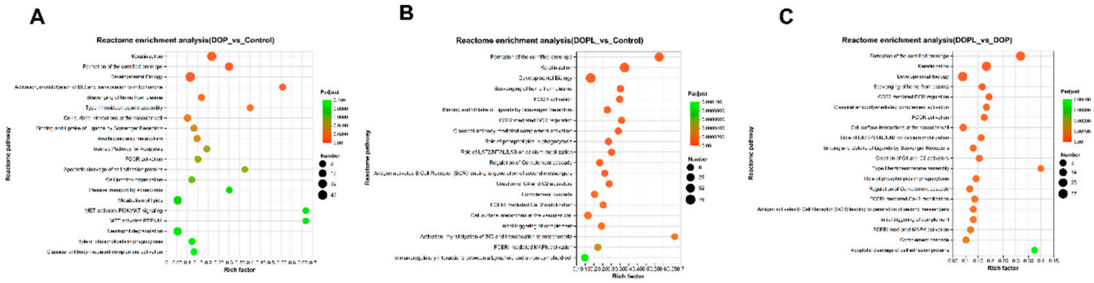

Figure S3. Reactome functional enrichment of DEGs in tumor tissues from different groups (n = 3).

1. Zhang, X.; Duan, S.; Tao, S.; Huang, J.; Liu, C.; Xing, S.; Ren, Z.; Lei, Z.; Li, Y.; Wei, G., Polysaccharides from Dendrobium officinale inhibit proliferation of osteosarcoma cells and enhance cisplatin-induced apoptosis. *J. Funct. Foods* **2020,** *73*, 104143.
2. Tao, S.; Song, Y.; Ding, S.; He, R.; Shi, Q.; Hu, F., Dendrobium officinale polysaccharide-based carrier to enhance photodynamic immunotherapy. *Carbohydr. Polym.* **2023,** *317*, 121089.
